# Survey of clustered regularly interspaced short palindromic repeats and their associated Cas proteins (CRISPR/Cas) systems in multiple sequenced strains of *Klebsiella pneumoniae*

**DOI:** 10.1186/s13104-015-1285-7

**Published:** 2015-08-04

**Authors:** Martha Lorena Ostria-Hernández, Carlos Javier Sánchez-Vallejo, J Antonio Ibarra, Graciela Castro-Escarpulli

**Affiliations:** Laboratorio de Bacteriología Médica y, Escuela Nacional de Ciencias Biológicas, Instituto Politécnico Nacional, Prolongación de Carpio y Plan de Ayala S/N, Colonia Santo Tomás, Delegación Miguel Hidalgo, CP 11340 Mexico, D.F., Mexico; Laboratorio de Genética Microbiana, Departamento de Microbiología, Escuela Nacional de Ciencias Biológicas, Instituto Politécnico Nacional, Prolongación de Carpio y Plan de Ayala S/N, Colonia Santo Tomás, Delegación Miguel Hidalgo, CP 11340 Mexico, D.F., Mexico; Departamento de Bioquímica, Laboratorio de Biotecnología y Bioinformática Genómica, Escuela Nacional de Ciencias Biológicas, Instituto Politécnico Nacional, Prolongación de Carpio y Plan de Ayala S/N, Colonia Santo Tomás, Delegación Miguel Hidalgo, CP 11340 Mexico, D.F., Mexico

**Keywords:** CRISPR/Cas, Bacterial immune system, Bacteriophages, Plasmids, Multiple drug resistance

## Abstract

**Background:**

In recent years the emergence of multidrug resistant *Klebsiella pneumoniae* strains has been an increasingly common event. This opportunistic species is one of the five main bacterial pathogens that cause hospital infections worldwide and multidrug resistance has been associated with the presence of high molecular weight plasmids. Plasmids are generally acquired through horizontal transfer and therefore is possible that systems that prevent the entry of foreign genetic material are inactive or absent. One of these systems is CRISPR/Cas. However, little is known regarding the clustered regularly interspaced short palindromic repeats and their associated Cas proteins (CRISPR/Cas) system in *K. pneumoniae.* The adaptive immune system CRISPR/Cas has been shown to limit the entry of foreign genetic elements into bacterial organisms and in some bacteria it has been shown to be involved in regulation of virulence genes. Thus in this work we used bioinformatics tools to determine the presence or absence of CRISPR/Cas systems in available *K. pneumoniae* genomes.

**Results:**

The complete CRISPR/Cas system was identified in two out of the eight complete *K. pneumoniae* genomes sequences and in four out of the 44 available draft genomes sequences. The *cas* genes in these strains comprises eight *cas* genes similar to those found in *Escherichiacoli*, suggesting they belong to the type I-E group, although their arrangement is slightly different. As for the CRISPR sequences, the average lengths of the direct repeats and spacers were 29 and 33 bp, respectively. BLAST searches demonstrated that 38 of the 116 spacer sequences (33%) are significantly similar to either plasmid, phage or genome sequences, while the remaining 78 sequences (67%) showed no significant similarity to other sequences. The region where the CRISPR/Cas systems were located is the same in all the *Klebsiella* genomes containing it, it has a syntenic architecture, and is located among genes encoding for proteins likely involved in metabolism and resistance to antibiotics.

**Conclusions:**

The CRISPR/Cas system is not widely distributed in *K. pneumoniae* genomes, those present most likely belong to type I-E with few differences from the arrangement of the *cse3* gene and most of the spacers have not been are not described yet. Given that the CRISPR/Cas system is scarcely distributed among *K. pneumoniae* genomes it is not clear whether it is involved in either immunity against foreign genetic material or virulence. We consider that this study represents a first step to understand the role of CRISPR/Cas in *K. pneumoniae*.

**Electronic supplementary material:**

The online version of this article (doi:10.1186/s13104-015-1285-7) contains supplementary material, which is available to authorized users.

## Background

During bacterial evolution, the ability of bacteria to adapt to new environments has been favored by the acquisition of genes through horizontal gene transfer (HGT) [1]. Despite this apparent advantage, each organism must balance the need to acquire beneficial characteristics through HGT with the need to prevent the entry of genetic elements that impose additional energy costs. A system that allows bacteria to limit the entry of genetic elements is the adaptive immune system CRISPR/Cas (clustered regularly interspaced short palindromic repeats and their associated Cas proteins), which has been described in many Bacteria and Archaea. Extensive research has shown that when a microorganism contains the CRISPR/Cas system in its genome, it survives initial infection by phages or plasmids because it can acquire a short DNA fragment, and then start up its machinery to recognize and degrade them. When the incoming DNA is harmful to the cell, e.g. lytic phages, having an active CRISPR/Cas system offers a selective advantage. In contrast, when external DNA is required for survival, e.g. against antibiotic-selective conditions, this inhibition can be detrimental [2, 3]. This prokaryotic immune system is based on the use of small RNAs that limit phage infection and the entrance of plasmids [2, 3]. The CRISPR elements are composed of small direct repeat sequences (DR) between 21 and 48 base pairs (bp), separated by hypervariable sequences or spacers that range in size from 26 to 72 bp. Many of these sequences are derived from mobile genetic elements such as plasmids and phages [2, 4, 5]. An important step in understanding the role of CRISPR was the identification of genes located very close to them and their conservation in the different CRISPR systems. These genes were called *cas* because of their association with the CRISPR repeats [4, 6]. It was determined that the Cas proteins contain domains characteristic of nucleases, helicases, polymerases and various RNA-binding proteins [7]. The number of *cas* genes may vary from 4 to over 20 and the product of these genes, the Cas proteins, provide the enzymatic machinery required for the acquisition of spacers as well as for invader element marking. The central Cas proteins are characterized by their proximity to the CRISPR loci and are widely distributed among bacterial and archaeal species. Experiments in multiple bacteria have shown that these systems play an important role in the exchange of genetic material and they may affect its evolution rate [8]. Additionally, the CRISPR/Cas expression is tightly regulated but the information is limited to some genera [3, 5, 8–13].

Furthermore, an additional function has recently been attributed to this system: That is regulation of gene expression related to virulence. This function has been observed in several pathogenic bacteria [8, 14, 15]. Some studies highlight the potential of the CRISPR/Cas system to modulate bacterial physiology and the unexpected forms that could mitigate or increase its virulence [14]. In this regard, in *Pseudomonas aeruginosa* this system is capable of modulating biofilm production, an important virulence factor for this and various other pathogenic microorganisms [15]; in *Streptococcus pyogenes* the presence of the CRISPR/Cas system modulates prophage contents and hence its virulence; in *Enterococcus faecalis* and *E. faecium* a correlation between the absence of CRISPR/Cas and the presence of antimicrobial resistance genes has been determined [14–16]. Lastly, in *Francisella*, RNA-RNA interactions (mediated by the Cas9 protein), result in a reduction of essential lipoprotein transcripts for the pro-inflammatory response of the host, consequently these bacteria persist and cause infection in animal models [14, 15]. For these reasons, the role of this system in modulating virulence and antibiotic resistance in different bacterial pathogens represents an excellent opportunity as a research area.

*Klebsiella pneumoniae* is among the bacteria that have emerged as important opportunistic pathogens in hospital environments due to their high rate of antibiotic resistance and high degree of dispersion [17, 18]. The multidrug resistance of this bacterium has been associated with the presence of high molecular weight plasmids [17, 19]. Despite the increasing information on the presence of CRISPR/Cas in several pathogens, information about these systems in *K. pneumoniae* is scarce. Therefore, the main goal of this study was to determine, by means of bioinformatics tools and manual curation, the presence or absence of the CRISPR/Cas system in available *K. pneumoniae* genomes. Our results show that the CRISPR/Cas system is not homogeneously distributed in these bacteria and in those where it is present it is inserted at the same genome site. Additionally, most DR sequences in the CRISPR elements are specific for *K. pneumoniae,* as no homologues were found.

## Results

### Survey of CRISPR/Cas system in *K. pneumoniae* genomes

Given that only a small number of *K. pneumoniae* genomes have been completely annotated and reported we used also completed but not assembled genomes in this study. In total, 52 complete and draft genomes of *K. pneumoniae* were analyzed for the presence of components of the CRISPR/Cas system by using the CRISPRFinder software [20]. This program was used with the genomes already loaded on its database and with draft sequences that were uploaded manually after BLAST searches were performed as described in the "Methods" section. CRISPR sequence arrays and *cas* genes were detected in two out of the eight complete genomes and in four out of the 44 draft genome sequences available (Table 1 and Additional file 1: Table S1). In some cases CRISPRFinder detected regions with CRISPR, such as strains 342 and JM45, but no adjacent *cas* genes were found. These sequences were not considered to have a CRISPR/Cas system and were not included in the subsequent analysis. In order to corroborate CRISPRFinder results, five 20 kbp random sequences derived from the strain 1084 were generated (see "Methods" section) and analyzed. These data showed that no CRISPR sequences were found in any of the sequences analyzed. Taken together these results demonstrate that the CRISPR/Cas system is not homogenously distributed in all *K. pneumoniae* strains and it was found in only 12% of the analyzed strains.

**Table 1:** *Klebsiella pneumoniae* strains with a CRISPR/Cas system

Strain	Origin	Genome status	Number of CRISPR^a^	Number of spacers^a^	GenBank access no.	References
*K. pneumoniae* subsp. *pneumoniae* 1084	Liver abscess	Complete	2	8 and 14	NC_018522	[21, 22]
*K. pneumoniae* NTUH-K2044	Liver abscess	Complete	2	3 and 22	NC_012731	[23]
*K. pneumoniae* JHCK1	Meningitis	Draft	2	9 and 15	NZ_ANGH02000012	[24, 25]
*K. pneumoniae* RYC492	Stool	Draft	1	11	NZ_APGM01000001	[26, 27]
*K. pneumoniae* WGLW2	Sputum	Draft	1	9	NZ_JH930419	NR
*K. pneumoniae* WGLW5	Mouse	Draft	2	6 and 19	NZ_JH930428	NR

*NR* no reference, *WGLW2 and WGLW5* genomes were submitted by Broad Institute, *BioProjects* PRJNA181874, PRJNA169454 and PRJNA181876, PRJNA169456 respectively, available in NCBI.

^a^CRISPR/Cas system was detected with the CRISPRFinder software.

### Genomic context of CRISPR/Cas

**Fig. 1:**
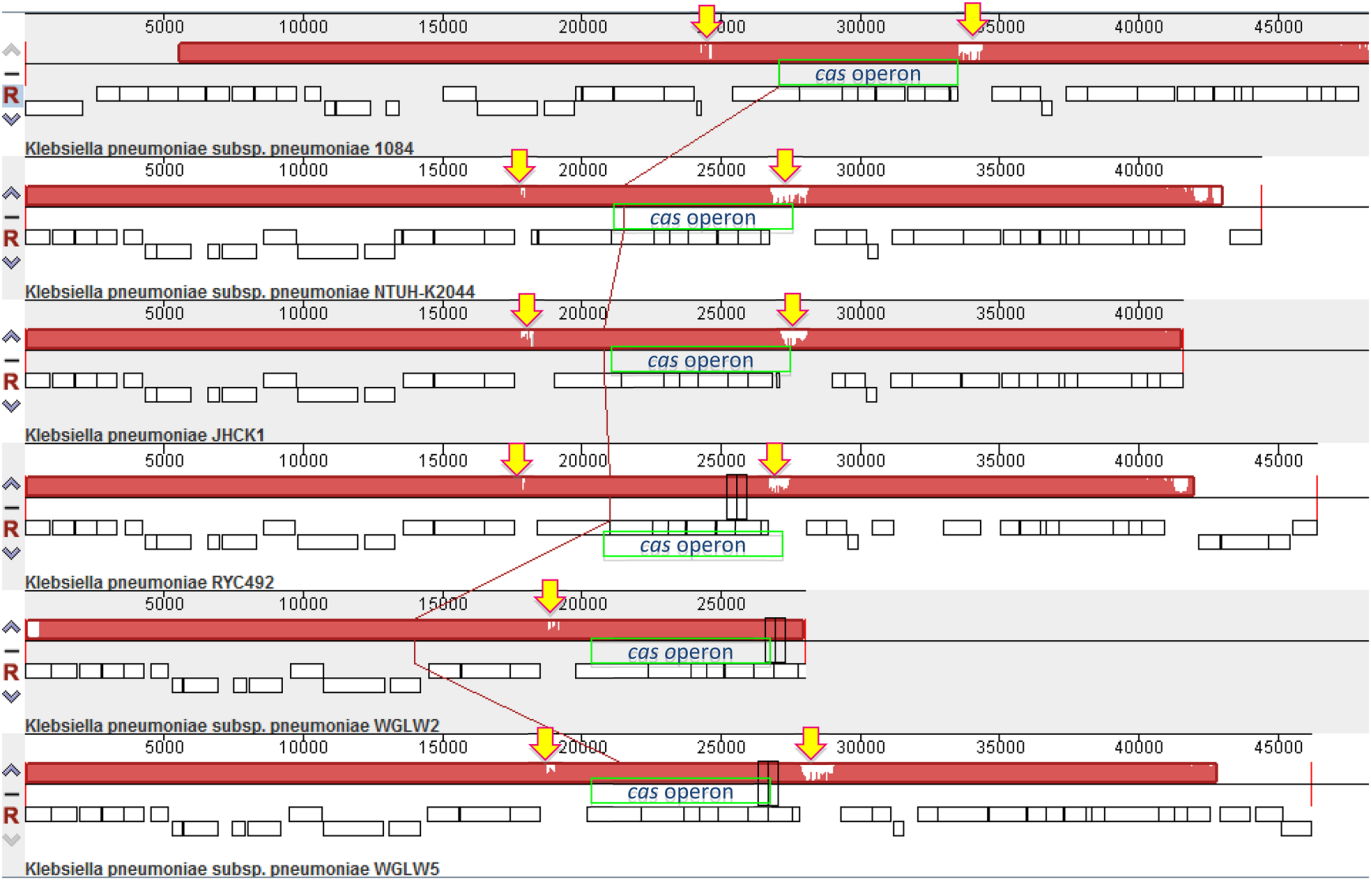
Location of CRISPR/Cas system in the genome of diverse strains of *Klebsiella pneumoniae*. Alignment generated with Progressive MAUVE of the six genomes that contain CRISPR/Cas. The region was grouped into a single locally collinear block (*red*). At the ends of the *cas* operon (*empty* or *blank regions* marked by *yellow arrows*), there is variability, probably due to the presence of CRISPR sequences.

In general, based on the MAUVE alignment (for details refer to "Methods" section) it was observed that the region where the *cas* operon is located in all genomes, only one locally collinear block (LCB) was found. Therefore, this region seems to be shared and syntenic. In the genomes of the strains NTUH-K2044 [GenBank: NC_012731], WGLW2 [GenBank: NZ_JH930419], and WGLW5 [GenBank: NZ_JH930428], this system was found encoded in the complementary strand. In contrast, in the genomes of strains 1084 [GenBank: NC_018522], JHCK1 [GenBank: NZ_ANGH02000012] and RYC492 [GenBank: NZ_APGM01000001], it was found encoded in the plus strand. We also found that upstream and downstream sequences of the *cas* operon were variable in all cases, which shows the variability of the CRISPR sequences (Fig. 1).

**Fig. 2:**
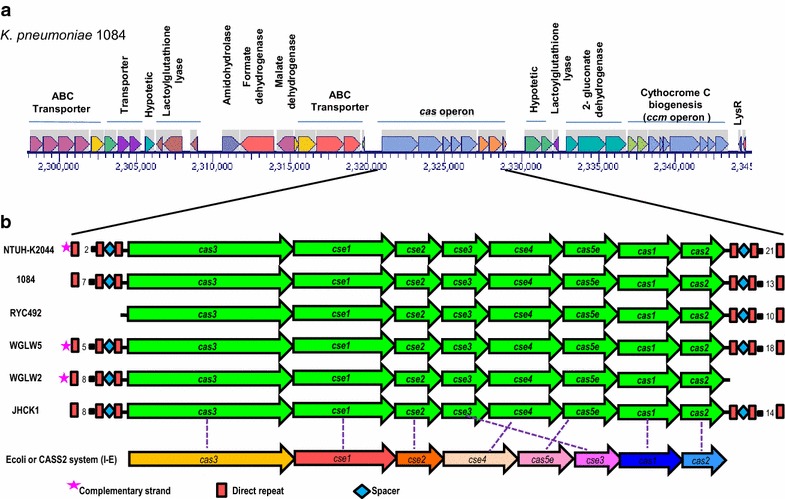
Genomic context of the CRISPR/Cas system in diverse strains of *Klebsiella pneumoniae.* **a** Genomic context of *cas* operon. Enzymes related to bacterial metabolism and some antibiotic resistance genes are located in the vicinity of cas operon. **b** CRISPR/Cas organization. The *cas* operon consists of eight genes and the CRISPR sequences are located downstream from *cas2* and upstream from *cas3* in those genomes containing two CRISPR arrays.

When analyzing the region upstream of the CRISPR/Cas we observed that genes were identical and encode for different subunits of an ABC type transporter (ID: AFQ65464, AFQ65465, AFQ65466), multiple subunits of a formate dehydrogenase (ID: AFQ65461), malate dehydrogenase (ID: AFQ65462), and amino acid transporters (ID: AFQ65453, AFQ65454, AFQ65460). Interestingly, we also found genes that seem to code proteins for antimicrobial resistance such as glyoxalase and efflux pumps (MdtM, multidrug efflux system protein) (ID: AFQ65457, AFQ65459, EMH97621). On the other hand, and similarly that observed at the 5′ end, at the 3′ end of the CRISPR/Cas region there was no variability. This region contains genes related to antibiotic resistance, such as lactoylglutathione lyase (or glyoxalase, which confers resistance to bleomycin) (ID: AFQ65478), and genes encoding different subunits of proteins involved in cell metabolism, such as 2-gluconate dehydrogenase (ID: AFQ65479, AFQ65480, AFQ65481), heme protein exporters (ID: BAH63785, BAH63784), and proteins involved in the biogenesis of cytochrome C (ID: AFQ63377, AFQ63378, AFQ65482, AFQ65483, AFQ65484, AFQ65485, AFQ65486, AFQ65487, AFQ65488) (Fig. 2a). In the draft genomes, most of the genes are annotated as hypothetical; however, a detailed analysis of this region reveals that the size and sequence of the genes is similar amongst all genomes with these systems. Taken together our analysis demonstrated that those *K. pneumoniae* strains harboring a CRISPR/Cas system are syntenic.

### Organization of the *cas* operon

As mentioned before, for the CRISPR/Cas systems there are always associated coding genes to the CRISPR sequences. *K. pneumoniae*, draft genomes and complete genomes, this system consists of eight *cas* genes that are syntenic (Fig. 2b). The *cas* genes identified were, from 5′ to 3′ direction: *cas3*, *cse1* also known as *casA*, *cse2* also known as *casB, cse3* or *casE, cse4* or *casC, cas5e, cas1* and *cas2.* As a whole, this suggests that the *cas* operon is conserved in those strains containing CRISPR/Cas systems and probably has a common evolutionary history in all these *Klebsiella* strains. This finding suggests that the cenancestor of *Klebsiella* contained the CRISPR system.

### Analysis of the CRISPR sequences

In all genomes containing the CRISPR/Cas system, CRISPR sequences were found upstream of the *cas3* gene and downstream of the *cas2* gene, in those genomes with two CRISPR arrays. Strains RYC492 and WGLW2, presented only one CRISPR array. In the RYC492 strain, the array was located downstream of *cas2* and contained 11spacers. In the WGLW2 genome the CRISPR sequence was upstream of *cas3* and had 3 spacers (Table 1; Fig. 2). Strains NTUH-K2044, 1084 and JHCK1 had two CRISPR arrays: The NTUH-K2044 strain contained 22 spacers in one array (downstream of *cas2*) and three in the upstream of *cas3;* strain 1084 presented 14 and 8 spacers (downstream of *cas2* and upstream of *cas3*), respectively, whereas strain JHCK1 contained 15 and 9 spacers (downstream of *cas2* and upstream of *cas3)*. The average length of the repeats was 29 bp whereas spacers had an average length of 33 bp.

**Fig. 3:**
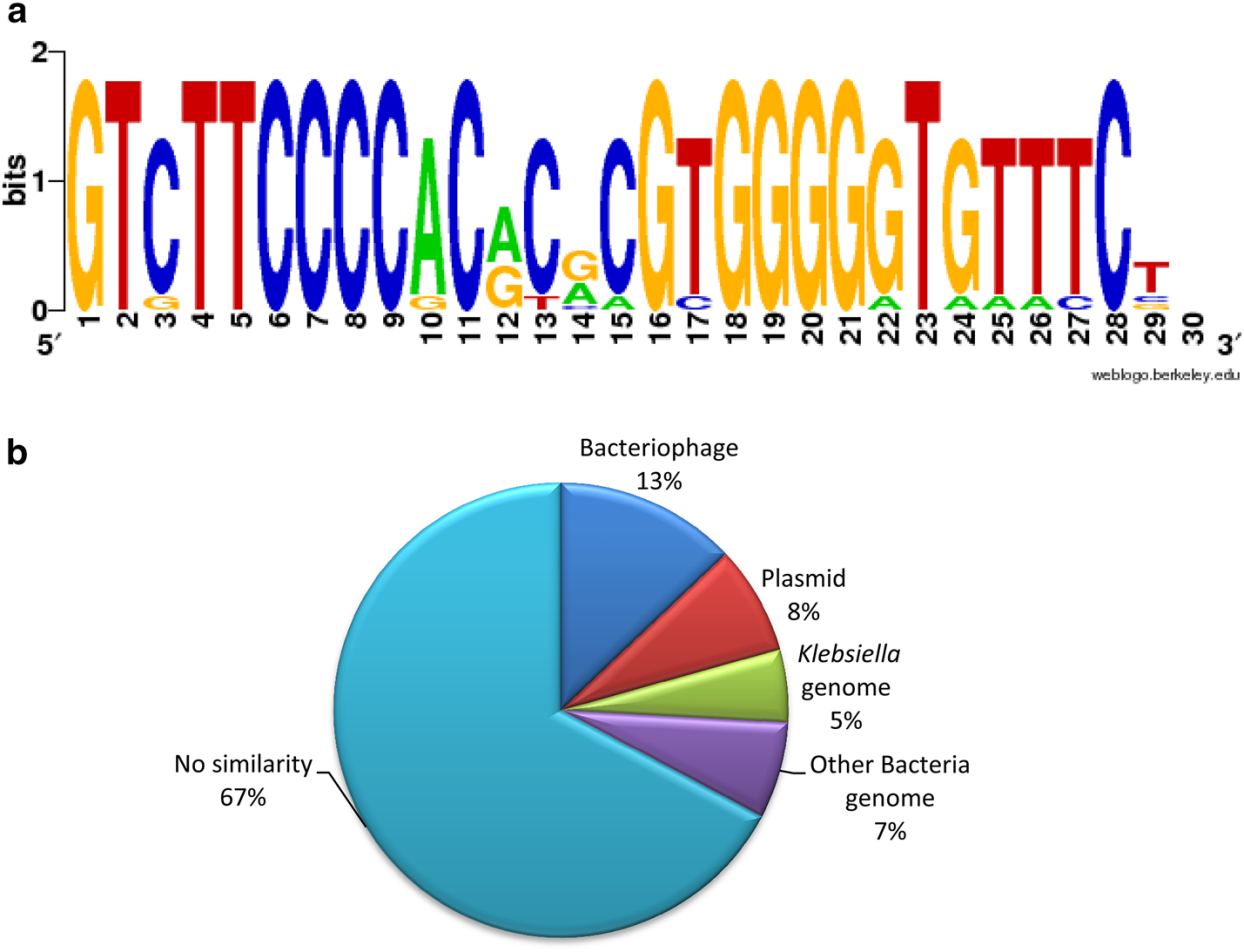
Description of direct repeats and spacer sequences found in *Klebsiella pneumoniae* genomes. **a** Logo obtained in WebLogo of the direct repeats consensus sequences of CRISPR arrays. The sequences are partially palindromic and symmetrical. **b** Match of spacer sequences with sequences of phages, plasmids and bacterial genomes deposited in GenBank.

Subsequently, and based on the comparison of the *cas* operon of *K. pneumoniae* with that of *E. coli* (Type I-E or CASS2), we observed that *K. pneumoniae* strains have the same number of genes but with a difference in the location of *cse3*. That is, for *E. coli cse3* is located downstream of *cas5e* while in *K. pneumoniae* it is located between *cse2* and *cse4* (Fig. 2). Whether this rearrangement influences the formation of the CASCADE complex involved in the recognition of foreign genetic material in *K. pneumoniae* still unknown and a matter of future research. In order to characterize the DRs in each CRISPR sequence we performed a detailed analysis by aligning all 10 of the DRs obtained through the analysis derived from the CRISPRFinder. The consensus sequence of these DRs showed a conserved GT(C/g)TTCCCC sequence at the 5′ region and a conserved GGGG(G/a)T(G/a)(T/a) (T/a)(T/c)C at the 3′ region. The main changes were detected in the middle of the sequence (position 12 to 15). Our results show that the DR sequence was symmetrical and partially palindromic (Fig. 3). Given the immune role exerted by the CRISPR/cas system, it has been observed that the spacer sequences are derived from HGT material [28]. In order to define the origin of the spacers in the systems identified in *K. pneumoniae* BLASTn searches were performed. This analysis showed that 38 of the 116 spacer sequences (33%) have significant similarity to plasmids, phages or genome sequences in *Klebsiella* or other bacteria. The distribution of these sequences was: 13% (15/116) of the spacer sequences had similarity to genes belonging to phages, 8% (9/116) corresponded to gene sequences of plasmids, 5% (6/116) to genes of the *Klebsiella* spp. genome, while 7% (8/116) were similar to genes that belong to genomes of other bacteria. The remaining 78 sequences (67%) showed no significant similarity to any other sequence (Fig. 3). In addition, strains that share spacer sequences were not detected. These results show a diverse origin of the CRISPR sequences, indicating that they were probably acquired from diverse events involving the entry of foreign genetic material.

## Discussion

The CRISPR/Cas systems are involved in limiting the entrance of foreign DNA to Bacteria and Archaea and have also been related to the expression of virulence factors in Bacteria. These systems have been widely studied in several organisms including pathogens and non-pathogens but few studies have demonstrated their in vivo activity [29]. Hence, and according to Bondy-Denomy and Davidson, 2014, the information on the content of spacers, the percentage of them presenting concordance with a known sequence or of those that are unique to a specific species, is hard to find in publications, and would provide a fundamental point of view on the functionality of this system. These data will be useful to interpret the functions of these systems in the strains being studied and will improve our understanding of bacterial evolution, as well as the impact of horizontal transfer of genes in the environment and human health [29]. To our understanding these systems have not been studied yet or characterized in *K. pneumoniae*. *K. pneumoniae* is among the top five pathogens causing nosocomial infections worldwide and it belongs to the ESKAPE group [30–35]*. K. pneumoniae* easily disperses in hospital wards, contains diverse virulence factors and has large plasmids conferring ecological advantages for its adaptation to several niches [17, 18]. We were therefore interested in knowing whether *K. pneumoniae* has CRISPR/Cas systems and whether or not these are related to HGT, multidrug resistance (MDR) or virulence. Here we used bioinformatics tools to determine the presence or absence of CRISPR/Cas systems in the available *K. pneumoniae* genomes. Our results revealed the presence of these systems in six out of 52 (8 complete and 44 draft) genomes available in databases. The latter suggests that CRISPR/Cas systems are not widely distributed in this bacterial species. Given the MDR characteristics and plasmid presence for all the analyzed strains shown in Additional file 1: Table S1 it is not possible to conclude whether the CRISPR/Cas system is related to either drug resistance or presence or absence of plasmids. Given that CRISPR/Cas systems are poorly distributed amongst *K. pneumoniae* strains they are not useful as a typing method as in other bacterial species. Determining whether these systems are related to MDR or to the presence of plasmids or phages requires further experimentation.

In the strains where the CRISPR/Cas system was found, it is located at the same site in all the genomes. The location of CRISPR/Cas systems is quite variable in other bacteria that have them for example in *Gardnerella vaginalis* it is located between *clpC* and the gene encoding for tRNA_Gly_ [36]; in *Campylobacter jejuni* (strain UPTC CF89-12) and *Campylobacter lari* (strain RM2100), the system is found between the *tmrU* structural genes and a PGPase [37]. In *E. coli* K12 and *S.Typhimurium* LT2, the system is found among metabolic genes. In our study we observed that in *K. pneumoniae* the system was located downstream from genes possibly encoding for subunits of an ABC transporter and upstream of a possible hemagglutinin. Based on our results and those described before it appears that the location of the CRISPR/Cas system differs depending on the bacteria, while it seems to be located at the same site in the genome among strains of the same species.

We also searched for *cas* genes closely related to the CRISPR arrays in those *K. pneumoniae* genomes containing the CRISPR/Cas system. In all cases we detected eight *cas* genes most likely forming an operon. Then we compared the organization of this *cas* operon to others already characterized. Regarding the number of genes and their organization this was similar to that of the *cas* operon in *E. coli* K12, except for one gene (*cse3*) located in a different position [7]. This suggests that the CRISPR/Cas system described here belong to the type I, subtype E group also known as CASS2 or Ecoli [7]. Included in this group are also the *cas* loci of *G. vaginalis* and *S.enterica*. This particular CRISPR/Cas system type has been detected frequently in pathogenic bacteria (37%), and at least in most of the enterobacteria in which it has been sought [7, 13–15]. Given that in some bacteria CRISPR/Cas systems are involved in virulence gene regulation, efforts to determine whether the CRISPR/Cas system in *K. pneumoniae* is involved in virulence should be conducted.

In regard with the CRISPR sequences, four out of the six systems detected in *K. pneumoniae* have two CRISPR arrays, one located upstream and one downstream of the *cas* operon. This contrasts with other CRISPR arrays in other bacteria, which usually have one located downstream of *cas2* [13, 36, 37]. Duplication of CRISPR sequences in these systems might have a role in the mechanisms involved in the acquisition and transcription of these arrays. The latter would have to be studied thoroughly and is a project that we are currently working on in our laboratory. Alternatively, the DR and spacers that are repeated in each CRISPR could be indicative of the “immune” memory of these bacteria. These findings highlight the importance of the research on CRISPR/Cas characterization in *K. pneumoniae*.

The length of the DR sequences of *K. pneumoniae* CRISPR arrays was 29–30 bp. In comparison, in *S.* Typhi these DR are 29 bp, in *G. vaginalis,* 28 bp and in *C. jejuni,* 36 bp. This is consistent with reports in the literature that indicate that these DR sequences may have a size between 21 to 48 bp [4, 13, 36, 37]. Concerning the nucleotide sequence of the CRISPR region, in *K. pneumoniae* the DR sequences were the same in each CRISPR array of a single strain but different from strain to strain, similar to what has been observed in other CRISPR arrays. On the other hand, this contrasts with reported on *S.* Typhi IMSS CRISPR-1 which DR sequences are similar to those in *S.* Typhi CT18 and *E. coli* K12, as well as in *C. jejuni* strain UPTC CF89-12 whose consensus sequence has a 92–100% similarity with repeats from other *Campylobacter* strains. At this moment we cannot predict whether the DR in *K. pneumoniae* is evolutionarily related to those in *E. coli* or not.

Regarding the spacer sequences, these are incorporated into the CRISPR array and provide a historical view of the exposure of bacteria to a variety of external genetic elements [38]. Spacer sequences in *K. pneumoniae* have an average size of 33 bp, similar to those reported in *G. vaginalis*, *C. jejuni*, and *S.* Typhi. This is consistent with reports in the literature indicating that these spacers can have a size between 26 and 72 bp [4, 13, 36, 37].

In this study, 38 out of 116 CRISPR spacer sequences showed similarities with either phage, plasmids or bacterial genomes. This characteristic has been widely reported in other spacer sequences and is one of the bases for the interest in these systems [28]. In our study we were able to detect that some of these sequences were similar to more than one region of the same phage or plasmid sequence but from a different region. Findings such as these suggest that CRISPR interference prevents phage or common plasmid acquisition in these strains and those different regions are recognized and included as spacers [8].

Furthermore, it has also been reported that spacer sequences have no similarity to other sequences of foreign genetic material in a variety of bacteria such as *S.* Typhi, *G. vaginalis* and *C. jejuni* [13, 36, 37]. However, in this study, the proportion of sequences that did show similarity to others contained in the GenBank database was greater than in any previous reports. The concordance of the detected spacer sequences was mainly with integrases, viral replication proteins or plasmids, gene sequences coding for viral structures, exonucleases, and hypothetical or non-described genes. Likewise, a similarity to *cas* gene sequences or the *Klebsiella* genome itself was found; this fact suggests what in some studies has been proposed as autoimmunity. This means that marked CRISPR/Cas system sequences cause a partial or total degradation of their own activity. Another possibility is that the incorporation of this type of spacers occurs due to errors of the Cas proteins involved in this process [2]. However, the mechanisms that select the regions that will integrate the CRISPR remain unknown, but it is suggested that they are not random events and that protospacer adjacent motifs, also called PAM (a sequence located at the 3′ or 5′ end of the protospacer or the sequence present in the foreign genetic element), seem to determine the orientation of the spacer within the repeat array [4].

## Conclusion

The search for and the description of this system in pathogenic bacteria could have countless implications, ranging from success in new therapeutic procedures, typing methods or determining the evolutionary role of these microorganisms to finally understanding the reasons why this system has been lost or shut down and the implications that this could have on the emergence of highly virulent pathogens. In *K. pneumoniae*, the CRISPR/Cas system was not homogeneously distributed; however, detection in some strains opens the possibility to diverse hypotheses about its functionality and regulatory mechanisms. It is necessary to demonstrate that strains that contain it, keep it functional, as well as to determine the relationship between its presence and extrinsic environmental factors or regulatory mechanisms involved in its expression. Thus, understanding the role of this system could provide information about the evolutionary history of this and other pathogens.

## Methods

### Searching for the CRISPR/Cas in *K. pneumoniae*

The complete genomes of eight *K. pneumoniae* contained in the CRISPRdb database were analyzed with the CRISPRFinder platform available at http://crispr.u-psud.fr [20]. This algorithm locates direct repeat sequences of 23–55 bp separated by variable sequences of a size no greater than 2.5 times or no less than 0.6 times the length of the repeated sequences (25–60 bp). When the algorithm detects at least three repeating regions that are exactly the same (in sequence and size), which are separated by variable sequences, it is considered a “confirmed CRISPR”. If the algorithm locates two repeats separated by a variable sequence, it establishes the status of a “questionable CRISPR”. For the present study we only considered those indicated by the program as “confirmed CRISPRs”. In addition, with this platform, we searched for *cas* genes in regions adjacent to CRISPR sequences.

In order to search for the CRISPR/Cas system in draft genomes we downloaded 44 *K. pneumoniae* draft genome sequences from the NCBI database in ftp://ftp.ncbi.nlm.nih.gov/genomes/Bacteria_DRAFT to build a local database using Standalone BLAST (BLASTn and BLASTp). Given that *cas1* is considered a genetic and universal marker for the CRISPR/Cas systems we used the sequences of *cas1* and the Cas1 protein from *K. pneumoniae* strain 1084 to perform the BLAST searches as described above. Expectation values (e-value) less than or equal to 0.0001 for *cas1* and 0.001 for the protein were considered significant as well as a coverage percentage of more than or equal to 80%. As a control we also analyzed the sequences of all eight complete genomes expecting the same results as those shown in the CRISPRFinder database. Fragments containing *cas1* and Cas1 were selected, uploaded and analyzed with CRISPRFinder using the same parameter as with the complete genomes. To corroborate the CRISPRFinder results we generated five 20 kbp random sequences derived from the genome of strain 1084 using RSAT [39] and analyzed them using the CRISPRFinder platform. Our results showed that no CRISPR sequences were found in any of the sequences analyzed.

### CRISPR/Cas genomic context

The BioCyc platform, http://biocyc.org [40], was used to visualize the genomic context of the *cas* operon in complete genomes. Subsequently, the same analysis was performed with draft genomes containing *cas1* using CRISPRFinder and using the information contained in GenBank. Progressive alignment was performed with MAUVE using the default parameters set by the program, to determine the similarity and synteny in the regions obtained [41].

### Analysis of spacers and direct repeats (DRs)

Once the genomes with a CRISPR sequences were analyzed with the CRISPRFinder platform the detected spacer and repeat sequences were obtained as output files. Each spacer sequence was compared to genome sequences of viruses (taxid: 10239) and Bacteria (taxid: 2) available at the NCBI database using nucleotide BLAST and the BLASTn algorithm. The criteria used to determine significant similarity were e-values less than or equal to 0.0001 and a score greater than 40. All spacer sequences were aligned with the parameters set by the MAFFT program [42] to determine whether or not a CRISPR sequence is shared with other CRISPR sequences (service available at: http://www.ebi.ac.uk/Tools/msa/mafft/).

Furthermore, the consensus sequences of the DR from each CRISPR were also obtained from CRISPRFinder and aligned with ClustalX2 [43] and MUSCLE [44]. Subsequently, these were analyzed with the WebLogo software [45] to establish nucleotides that are conserved among all sequences.

## Additional file

Additional file 1:
**Table S1.** Analyzed *K. pneumoniae* genomes.

## Abbreviations

CRISPR: clustered regularly interspaced short palindromic repeats
CRISPR/Cas: CRISPR elements and their associated Cas proteins
HGT: horizontal gene transfer
MDR: multidrug resistance
