# Supplementary material for: Survey of clustered regularly interspaced short palindromic repeats and their associated Cas proteins (CRISPR/Cas) systems in multiple sequenced strains of Klebsiella pneumoniae
Source: BMC Res Notes. 2015 Aug 4;8:332. doi: 10.1186/s13104-015-1285-7 (PMC4522967; doi:10.1186/s13104-015-1285-7)
Supplement: Additional file 1: — Table S1. Analyzed K. pneumoniae genomes. [file 13104_2015_1285_MOESM1_ESM.doc]

Supplementary material

**Table S1.** Analyzed *K. pneumoniae* genomes

| **Draft genomes (scaffold)** | **Accession number** | **Features** | **REF** |
| --- | --- | --- | --- |
| Klebsiella pneumoniae_12_3578_uid199972 | NZ_AQOC00000000 | Isolated from blood of a patient with pneumonia | [1] |
| Klebsiella pneumoniae_700603_uid195436 | NZ_AQOG00000000 | MDR*, ESBL** producer | [2] |
| Klebsiella pneumoniae_ATCC_BAA_1705_uid195437 | NZ_AOGQ00000000 | KPC. MDR*** | [3] |
| Klebsiella pneumoniae_ATCC_BAA_2146_uid195435 | NZ_AOCV00000000 | MDR, NDM-1^§^ | [4] |
| Klebsiella pneumoniae_ATCC_BAA_2146_uid199969 | NZ_APNN00000000 | MDR  NDM-1 | [4] |
| Klebsiella pneumoniae_DSM_30104_uid180406 | NZ_AJJI00000000 | MDR | [[5]](http://dx.doi.org/10.1128%2FJB.01388-12) |
| Klebsiella pneumoniae_Ecl8_uid182102 | NZ_CANH00000000 | Streptomycin-resistant mutant. Genetically tractable for targeted gene deletion strategies.  1 plasmid | [6] |
| Klebsiella pneumoniae_HSL4_uid199961 | NZ_APFG00000000 | Isolated from mangrove sediment slurry | [7] |
| Klebsiella pneumoniae_JHCK1_uid1983378 | NZ_ANGH00000000 | MDR  5 plasmids | [8] |
| Klebsiella pneumoniae_KPNIH10_uid180470 | NZ_AKAC00000000 | KPC, MDR | [9] |
| Klebsiella pneumoniae_KPNIH11_uid180471 | NZ_AKAD00000000 | KPC, MDR | [9] |
| Klebsiella pneumoniae_KPNIH12_uid180472 | NZ_AKAE00000000 | KPC, MDR | [9] |
| Klebsiella pneumoniae_KPNIH14_uid180473 | NZ_AKAF00000000 | KPC, MDR | [9] |
| Klebsiella pneumoniae_KPNIH16_uid180474 | NZ_AKAG00000000 | KPC, MDR | [9] |
| Klebsiella pneumoniae_KPNIH17_uid180475 | NZ_AKAH00000000 | KPC, MDR | [9] |
| Klebsiella pneumoniae_KPNIH18_uid180476 | NZ_AKAI00000000 | KPC, MDR | [9] |
| Klebsiella pneumoniae_KPNIH19_uid180477 | NZ_AKAJ00000000 | KPC, MDR | [9] |
| Klebsiella pneumoniae_KPNIH1_uid180462 | NZ_AJZU00000000 | KPC, MDR | [9] |
| Klebsiella pneumoniae_KPNIH20_uid180478 | NZ_AKAK00000000 | KPC, MDR | [9] |
| Klebsiella pneumoniae_KPNIH21_uid180479 | NZ_AKAL00000000 | KPC, MDR | [9] |
| Klebsiella pneumoniae_KPNIH22_uid180480 | NZ_AKAM00000000 | KPC, MDR | [9] |
| Klebsiella pneumoniae_KPNIH23_uid180481 | NZ_AKAN00000000 | KPC, MDR | [9] |
| Klebsiella pneumoniae_KPNIH2_uid180463 | NZ_AJZV00000000 | KPC, MDR | [9] |
| Klebsiella pneumoniae_KPNIH4_uid180464 | NZ_AJZW00000000 | KPC, MDR | [9] |
| Klebsiella pneumoniae_KPNIH5_uid180465 | NZ_AJZX00000000 | KPC, MDR | [9] |
| Klebsiella pneumoniae_KPNIH6_uid180466 | NZ_AJZY00000000 | KPC, MDR | [9] |
| Klebsiella pneumoniae_KPNIH7_uid180467 | NZ_AJZZ00000000 | KPC, MDR | [9] |
| Klebsiella pneumoniae_KPNIH8_uid180468 | NZ_AKAA00000000 | KPC, MDR | [9] |
| Klebsiella pneumoniae_KPNIH9_uid180469 | NZ_AKAB00000000 | KPC, MDR | [9] |
| Klebsiella pneumoniae_KpO3210_uid199803 | NZ_AMRH00000000 | KPC  Resistant to all beta-lactams | [10] |
| Klebsiella pneumoniae_KpQ3_uid182046 | NZ_AMSU00000000 | MDR  1 Plasmid | [7] |
| Klebsiella pneumoniae_LCT_KP214_uid200061 | NZ_AJHE00000000 | MDR  3 plasmids | [11] |
| Klebsiella pneumoniae_LZ_uid200130 | NZ_AJVY00000000 | It produces 1,3-propanediol from glycerol. Isolated from soil | [12] |
| Klebsiella pneumoniae_PR04_uid199936 | NZ_AOPN00000000 | Isolated from a patient. | [13] |
| Klebsiella pneumoniae_RYC492_uid193790 | NZ_APGM01000001 | Microcin producer  MDR | [14] |
| Klebsiella pneumoniae_ST258_K26BO_uid180983 | NZ_CANR00000000 | MDR | [7] |
| Klebsiella pneumoniae_ST258_K28BO_uid180984 | NZ_CANS00000000 | MDR | [7] |
| Klebsiella pneumoniae_ST512_K30BO_uid180981 | NZ_CAJM00000000 | MDR | [7] |
| Klebsiella pneumoniae_VA360_uid183379 | NZ_ANGI00000000 | KPC, MDR  5 plasmids | [8] |
| Klebsiella pneumoniae_WGLW1_uid181873 | NZ_AMLL00000000 | Cultured from urine | [15] |
| Klebsiella pneumoniae_WGLW2_uid181874 | NZ_AMLM00000000 | Cultured from sputum | [15] |
| Klebsiella pneumoniae_WGLW3_uid181875 | NZ_AMLN00000000 | Cultured from stool | [15] |
| Klebsiella pneumoniae_WGLW5_uid181876 | NZ_AMLO00000000 | Mouse strain, cultured from stool | [15] |
| Klebsiella pneumoniae_hvKP1_uid189025 | NZ_AOIZ00000000.1 | Isolated from a hospitalized patient in Buffalo, New York | [7] |
| **Complete genomes (refseq)** | **Accession number** | **Features** | **REF** |
| Klebsiella pneumoniae_1084_uid174151 | NC_018522 | No plasmids  No ESBL producer | [16] |
| Klebsiella pneumoniae_342_uid59145 | NC_011283 | Nitrogen-fixing endophyte.  2 Plasmids | [17] |
| Klebsiella pneumoniae_KCTC_2242_uid162147 | NC_017540 | It produces 2,3-butanediol  1 Plasmid | [[18]](http://dx.doi.org/10.1128%2FJB.00027-12) |
| Klebsiella pneumoniae_NTUH_K2044_uid59073 | NC_012731 | 1 Plasmid  High virulence and hypermucoviscosity | [[19]](http://dx.doi.org/10.1128%2FJB.00315-09) |
| Klebsiella pneumoniae_HS11286_uid84387 | NC_016845 | MDR  6 Plasmids | [20] |
| Klebsiella pneumoniae_MGH78578_uid57619 | NC_009648 | 5 Plasmids | [7] |
| Klebsiella pneumoniae_CG43_uid223021 | [NC_022566.1](http://www.ncbi.nlm.nih.gov/nuccore/550443072) | Clinical isolate | [7] |
| Klebsiella pneumoniae_JM45_uid215235 | NC_022082 | Clinical isolate  Carbapenem resistance  2 plasmids | [1] |

*. MDR: multidrug resistant; **. ESBL, ***. KPC: *K. pneumoniae* carbapenemase producer.

^§^. NDM-1: Metallo-beta lactamase producer

**References**

# 1. http://ftp.ncbi.nlm.nih.gov/

# 2. [**Broberg CA**](http://www.ncbi.nlm.nih.gov/pubmed?term=Broberg CA%5BAuthor%5D&cauthor=true&cauthor_uid=23723407), [**Palacios M**](http://www.ncbi.nlm.nih.gov/pubmed?term=Palacios M%5BAuthor%5D&cauthor=true&cauthor_uid=23723407), [**Miller VL**](http://www.ncbi.nlm.nih.gov/pubmed?term=Miller VL%5BAuthor%5D&cauthor=true&cauthor_uid=23723407): Whole-Genome draft sequences of three multidrug-resistant *Klebsiella* *pneumoniae* strains available from the American Type Culture Collection. [***Genome Announc***](http://www.ncbi.nlm.nih.gov/pubmed/?term=10.1128%2FgenomeA.00312-13)2013, 3:pii: e00312-13.

# 3. **[www.atcc.org/products/all/BAA-1705.aspx#characteristics](http://www.atcc.org/products/all/BAA-1705.aspx#characteristics)**

# 4. www.atcc.org/products/all/BAA-1705.aspx#characteristics

# 5. [**Lee JH**](http://www.ncbi.nlm.nih.gov/pubmed?term=Lee JH%5BAuthor%5D&cauthor=true&cauthor_uid=23012294), [**Cheon IS**](http://www.ncbi.nlm.nih.gov/pubmed?term=Cheon IS%5BAuthor%5D&cauthor=true&cauthor_uid=23012294), [**Shim BS**](http://www.ncbi.nlm.nih.gov/pubmed?term=Shim BS%5BAuthor%5D&cauthor=true&cauthor_uid=23012294), [**Kim DW**](http://www.ncbi.nlm.nih.gov/pubmed?term=Kim DW%5BAuthor%5D&cauthor=true&cauthor_uid=23012294), [**Kim SW**](http://www.ncbi.nlm.nih.gov/pubmed?term=Kim SW%5BAuthor%5D&cauthor=true&cauthor_uid=23012294), [**Chun J**](http://www.ncbi.nlm.nih.gov/pubmed?term=Chun J%5BAuthor%5D&cauthor=true&cauthor_uid=23012294), [**Song M**](http://www.ncbi.nlm.nih.gov/pubmed?term=Song M%5BAuthor%5D&cauthor=true&cauthor_uid=23012294): Draft genome sequence of *Klebsiella pneumoniae* subsp. *pneumoniae* DSM 30104T. [***J Bacteriol***](http://www.ncbi.nlm.nih.gov/pubmed/?term=10.1128%2FJB.01388-12)2012, 20:5722-3

# 6. [**Fookes M**](http://www.ncbi.nlm.nih.gov/pubmed?term=Fookes M%5BAuthor%5D&cauthor=true&cauthor_uid=23405357), [**Yu J**](http://www.ncbi.nlm.nih.gov/pubmed?term=Yu J%5BAuthor%5D&cauthor=true&cauthor_uid=23405357), [**De Majumdar S**](http://www.ncbi.nlm.nih.gov/pubmed?term=De Majumdar S%5BAuthor%5D&cauthor=true&cauthor_uid=23405357), [**Thomson N**](http://www.ncbi.nlm.nih.gov/pubmed?term=Thomson N%5BAuthor%5D&cauthor=true&cauthor_uid=23405357), [**Schneiders T**](http://www.ncbi.nlm.nih.gov/pubmed?term=Schneiders T%5BAuthor%5D&cauthor=true&cauthor_uid=23405357): Genome sequence of *Klebsiella pneumoniae* Ecl8, a reference strain for targeted genetic manipulation. [***Genome Announc***](http://www.ncbi.nlm.nih.gov/pubmed/?term=10.1128%2FgenomeA.00027-12)2013, 1:pii: e00027-12

7. http://www.ncbi.nlm.nih.gov/genome/browse/

8. Xie G, Ramirez MS, Marshall SH, Hujer KM, Lo CC, Johnson S, Li PE, Davenport K, Endimiani A, Bonomo RA, Tolmasky ME, Chain PS: **Genome sequences of two *Klebsiella pneumoniae* isolates from different geographical regions, Argentina (strain JHCK1) and the United States (strain VA360).** *Genome Announc* 2013, **1**:e00168-13

# 9. [**Snitkin ES**](http://www.ncbi.nlm.nih.gov/pubmed?term=Snitkin ES%5BAuthor%5D&cauthor=true&cauthor_uid=22914622), [**Zelazny AM**](http://www.ncbi.nlm.nih.gov/pubmed?term=Zelazny AM%5BAuthor%5D&cauthor=true&cauthor_uid=22914622), [**Thomas PJ**](http://www.ncbi.nlm.nih.gov/pubmed?term=Thomas PJ%5BAuthor%5D&cauthor=true&cauthor_uid=22914622), [**Stock F**](http://www.ncbi.nlm.nih.gov/pubmed?term=Stock F%5BAuthor%5D&cauthor=true&cauthor_uid=22914622), [**NISC Comparative Sequencing Program Group**](http://www.ncbi.nlm.nih.gov/pubmed?term=NISC Comparative Sequencing Program Group%5BCorporate Author%5D), [**Henderson DK**](http://www.ncbi.nlm.nih.gov/pubmed?term=Henderson DK%5BAuthor%5D&cauthor=true&cauthor_uid=22914622), [**Palmore TN**](http://www.ncbi.nlm.nih.gov/pubmed?term=Palmore TN%5BAuthor%5D&cauthor=true&cauthor_uid=22914622), [**Segre JA**](http://www.ncbi.nlm.nih.gov/pubmed?term=Segre JA%5BAuthor%5D&cauthor=true&cauthor_uid=22914622): Tracking a hospital outbreak of carbapenem-resistant *Klebsiella pneumoniae* with whole-genome sequencing. [***Sci Transl Med***](http://www.ncbi.nlm.nih.gov/pubmed?term=10.1126/scitranslmed.3004129&cmd=correctspelling) 2012, 148:148ra116.

# 10. [**Wesselink JJ**](http://www.ncbi.nlm.nih.gov/pubmed?term=Wesselink JJ%5BAuthor%5D&cauthor=true&cauthor_uid=23209233), [**López-Camacho E**](http://www.ncbi.nlm.nih.gov/pubmed?term=López-Camacho E%5BAuthor%5D&cauthor=true&cauthor_uid=23209233), [**de la Peña S**](http://www.ncbi.nlm.nih.gov/pubmed?term=de la Peña S%5BAuthor%5D&cauthor=true&cauthor_uid=23209233), [**Ramos-Ruiz R**](http://www.ncbi.nlm.nih.gov/pubmed?term=Ramos-Ruiz R%5BAuthor%5D&cauthor=true&cauthor_uid=23209233), [**Ruiz-Carrascoso G**](http://www.ncbi.nlm.nih.gov/pubmed?term=Ruiz-Carrascoso G%5BAuthor%5D&cauthor=true&cauthor_uid=23209233), [**Lusa-Bernal S**](http://www.ncbi.nlm.nih.gov/pubmed?term=Lusa-Bernal S%5BAuthor%5D&cauthor=true&cauthor_uid=23209233), [**Fernández-Soria VM**](http://www.ncbi.nlm.nih.gov/pubmed?term=Fernández-Soria VM%5BAuthor%5D&cauthor=true&cauthor_uid=23209233), [**Gómez-Gil R**](http://www.ncbi.nlm.nih.gov/pubmed?term=Gómez-Gil R%5BAuthor%5D&cauthor=true&cauthor_uid=23209233), [**Gomez-Puertas P**](http://www.ncbi.nlm.nih.gov/pubmed?term=Gomez-Puertas P%5BAuthor%5D&cauthor=true&cauthor_uid=23209233), [**Mingorance J**](http://www.ncbi.nlm.nih.gov/pubmed?term=Mingorance J%5BAuthor%5D&cauthor=true&cauthor_uid=23209233): Genome sequence of OXA-48 carbapenemase-producing *Klebsiella pneumoniae* KpO3210. [***J Bacteriol.***](http://www.ncbi.nlm.nih.gov/pubmed/?term=10.1128%2FJB.01897-12)2012 24:6981

11. [Guo Y](http://www.ncbi.nlm.nih.gov/pubmed?term=Guo Y%5BAuthor%5D&cauthor=true&cauthor_uid=25015528), [Li Y](http://www.ncbi.nlm.nih.gov/pubmed?term=Li Y%5BAuthor%5D&cauthor=true&cauthor_uid=25015528), [Su L](http://www.ncbi.nlm.nih.gov/pubmed?term=Su L%5BAuthor%5D&cauthor=true&cauthor_uid=25015528), [Chang D](http://www.ncbi.nlm.nih.gov/pubmed?term=Chang D%5BAuthor%5D&cauthor=true&cauthor_uid=25015528), [Liu W](http://www.ncbi.nlm.nih.gov/pubmed?term=Liu W%5BAuthor%5D&cauthor=true&cauthor_uid=25015528), [Wang T](http://www.ncbi.nlm.nih.gov/pubmed?term=Wang T%5BAuthor%5D&cauthor=true&cauthor_uid=25015528), [Yuan Y](http://www.ncbi.nlm.nih.gov/pubmed?term=Yuan Y%5BAuthor%5D&cauthor=true&cauthor_uid=25015528), [Fang X](http://www.ncbi.nlm.nih.gov/pubmed?term=Fang X%5BAuthor%5D&cauthor=true&cauthor_uid=25015528), [Wang J](http://www.ncbi.nlm.nih.gov/pubmed?term=Wang J%5BAuthor%5D&cauthor=true&cauthor_uid=25015528), [Li T](http://www.ncbi.nlm.nih.gov/pubmed?term=Li T%5BAuthor%5D&cauthor=true&cauthor_uid=25015528), [Fang C](http://www.ncbi.nlm.nih.gov/pubmed?term=Fang C%5BAuthor%5D&cauthor=true&cauthor_uid=25015528), [Dai W](http://www.ncbi.nlm.nih.gov/pubmed?term=Dai W%5BAuthor%5D&cauthor=true&cauthor_uid=25015528), [Liu C](http://www.ncbi.nlm.nih.gov/pubmed?term=Liu C%5BAuthor%5D&cauthor=true&cauthor_uid=25015528): **Comparative genomic analysis of *Klebsiella pneumonia* (LCT-KP214) and a mutant strain (LCT-KP289) obtained after spaceflight.** [*BMC Genomics*](http://www.ncbi.nlm.nih.gov/pubmed/?term=comparative+genomic+analysis+of+Klebsiella+pneumonie+(LCT-KP214)) 2014, **15**:589

# 12. [**Tao F**](http://www.ncbi.nlm.nih.gov/pubmed?term=Tao F%5BAuthor%5D&cauthor=true&cauthor_uid=22843590), [**Tai C**](http://www.ncbi.nlm.nih.gov/pubmed?term=Tai C%5BAuthor%5D&cauthor=true&cauthor_uid=22843590), [**Liu Z**](http://www.ncbi.nlm.nih.gov/pubmed?term=Liu Z%5BAuthor%5D&cauthor=true&cauthor_uid=22843590), [**Wang A**](http://www.ncbi.nlm.nih.gov/pubmed?term=Wang A%5BAuthor%5D&cauthor=true&cauthor_uid=22843590), [**Wang Y**](http://www.ncbi.nlm.nih.gov/pubmed?term=Wang Y%5BAuthor%5D&cauthor=true&cauthor_uid=22843590), [**Li L**](http://www.ncbi.nlm.nih.gov/pubmed?term=Li L%5BAuthor%5D&cauthor=true&cauthor_uid=22843590), [**Gao C**](http://www.ncbi.nlm.nih.gov/pubmed?term=Gao C%5BAuthor%5D&cauthor=true&cauthor_uid=22843590), [**Ma C**](http://www.ncbi.nlm.nih.gov/pubmed?term=Ma C%5BAuthor%5D&cauthor=true&cauthor_uid=22843590), [**Xu P**](http://www.ncbi.nlm.nih.gov/pubmed?term=Xu P%5BAuthor%5D&cauthor=true&cauthor_uid=22843590): Genome sequence of *Klebsiella pneumoniae* LZ, a potential platform strain for 1,3-propanediol production. [***J Bacteriol***](http://www.ncbi.nlm.nih.gov/pubmed/?term=10.1128%2FJB.00864-12)2012, 16:4457-8.

13. [Zulkifli MH](http://www.ncbi.nlm.nih.gov/pubmed?term=Zulkifli MH%5BAuthor%5D&cauthor=true&cauthor_uid=23950113), [Teh LK](http://www.ncbi.nlm.nih.gov/pubmed?term=Teh LK%5BAuthor%5D&cauthor=true&cauthor_uid=23950113), [Lee LS](http://www.ncbi.nlm.nih.gov/pubmed?term=Lee LS%5BAuthor%5D&cauthor=true&cauthor_uid=23950113), [Zakaria ZA](http://www.ncbi.nlm.nih.gov/pubmed?term=Zakaria ZA%5BAuthor%5D&cauthor=true&cauthor_uid=23950113), [Salleh MZ](http://www.ncbi.nlm.nih.gov/pubmed?term=Salleh MZ%5BAuthor%5D&cauthor=true&cauthor_uid=23950113): **Draft genome sequence of *Klebsiella pneumoniae* isolate PR04.** [*Genome Announc*](http://www.ncbi.nlm.nih.gov/pubmed/?term=10.1128%2FgenomeA.00418-13) 2013, **4** pii: e00418-13.

# 14. Marcoleta A, Gutiérrez-Cortez S, Maturana D, Monasterio O, Lagos R: Whole-genome sequence of the microcin E492-producing strain *Klebsiella pneumoniae* RYC492. *Genome Announc* 2013, 1:e00178-13.

# 15. http://www.broadinstitute.org/annotation/genome/Klebsiella_group/GenomeDescriptions.html

16. [Lin AC](http://www.ncbi.nlm.nih.gov/pubmed?term=Lin AC%5BAuthor%5D&cauthor=true&cauthor_uid=23105059), [Liao TL](http://www.ncbi.nlm.nih.gov/pubmed?term=Liao TL%5BAuthor%5D&cauthor=true&cauthor_uid=23105059), [Lin YC](http://www.ncbi.nlm.nih.gov/pubmed?term=Lin YC%5BAuthor%5D&cauthor=true&cauthor_uid=23105059), [Lai YC](http://www.ncbi.nlm.nih.gov/pubmed?term=Lai YC%5BAuthor%5D&cauthor=true&cauthor_uid=23105059), [Lu MC](http://www.ncbi.nlm.nih.gov/pubmed?term=Lu MC%5BAuthor%5D&cauthor=true&cauthor_uid=23105059), [Chen YT](http://www.ncbi.nlm.nih.gov/pubmed?term=Chen YT%5BAuthor%5D&cauthor=true&cauthor_uid=23105059): [**Complete genome sequence of *Klebsiella pneumoniae* 1084, a hypermucoviscosity-negative K1 clinical strain**](http://www.ncbi.nlm.nih.gov/pmc/articles/PMC3486373/)**.** *J Bacteriol* 2012, **194**: 6316.

17. [Fouts DE](http://www.ncbi.nlm.nih.gov/pubmed?term=Fouts DE%5BAuthor%5D&cauthor=true&cauthor_uid=18654632), [Tyler HL](http://www.ncbi.nlm.nih.gov/pubmed?term=Tyler HL%5BAuthor%5D&cauthor=true&cauthor_uid=18654632), [DeBoy RT](http://www.ncbi.nlm.nih.gov/pubmed?term=DeBoy RT%5BAuthor%5D&cauthor=true&cauthor_uid=18654632), [Daugherty S](http://www.ncbi.nlm.nih.gov/pubmed?term=Daugherty S%5BAuthor%5D&cauthor=true&cauthor_uid=18654632), [Ren Q](http://www.ncbi.nlm.nih.gov/pubmed?term=Ren Q%5BAuthor%5D&cauthor=true&cauthor_uid=18654632), [Badger JH](http://www.ncbi.nlm.nih.gov/pubmed?term=Badger JH%5BAuthor%5D&cauthor=true&cauthor_uid=18654632), [Durkin AS](http://www.ncbi.nlm.nih.gov/pubmed?term=Durkin AS%5BAuthor%5D&cauthor=true&cauthor_uid=18654632), [Huot H](http://www.ncbi.nlm.nih.gov/pubmed?term=Huot H%5BAuthor%5D&cauthor=true&cauthor_uid=18654632), [Shrivastava S](http://www.ncbi.nlm.nih.gov/pubmed?term=Shrivastava S%5BAuthor%5D&cauthor=true&cauthor_uid=18654632), [Kothari S](http://www.ncbi.nlm.nih.gov/pubmed?term=Kothari S%5BAuthor%5D&cauthor=true&cauthor_uid=18654632), [Dodson RJ](http://www.ncbi.nlm.nih.gov/pubmed?term=Dodson RJ%5BAuthor%5D&cauthor=true&cauthor_uid=18654632), [Mohamoud Y](http://www.ncbi.nlm.nih.gov/pubmed?term=Mohamoud Y%5BAuthor%5D&cauthor=true&cauthor_uid=18654632), [Khouri H](http://www.ncbi.nlm.nih.gov/pubmed?term=Khouri H%5BAuthor%5D&cauthor=true&cauthor_uid=18654632), [Roesch LF](http://www.ncbi.nlm.nih.gov/pubmed?term=Roesch LF%5BAuthor%5D&cauthor=true&cauthor_uid=18654632), [Krogfelt KA](http://www.ncbi.nlm.nih.gov/pubmed?term=Krogfelt KA%5BAuthor%5D&cauthor=true&cauthor_uid=18654632), [Struve C](http://www.ncbi.nlm.nih.gov/pubmed?term=Struve C%5BAuthor%5D&cauthor=true&cauthor_uid=18654632), [Triplett EW](http://www.ncbi.nlm.nih.gov/pubmed?term=Triplett EW%5BAuthor%5D&cauthor=true&cauthor_uid=18654632), [Methé BA](http://www.ncbi.nlm.nih.gov/pubmed?term=Methé BA%5BAuthor%5D&cauthor=true&cauthor_uid=18654632): **Complete genome sequence of the N2-fixing broad host range endophyte *Klebsiella pneumoniae* 342 and virulence predictions verified in mice.** [*PLoS Genet*](http://www.ncbi.nlm.nih.gov/pubmed/?term=10.1371%2Fjournal.pgen.1000141) 2008, **7**:e1000141.

18. [Shin SH](http://www.ncbi.nlm.nih.gov/pubmed?term=Shin SH%5BAuthor%5D&cauthor=true&cauthor_uid=22535926), [Kim S](http://www.ncbi.nlm.nih.gov/pubmed?term=Kim S%5BAuthor%5D&cauthor=true&cauthor_uid=22535926), [Kim JY](http://www.ncbi.nlm.nih.gov/pubmed?term=Kim JY%5BAuthor%5D&cauthor=true&cauthor_uid=22535926), [Lee S](http://www.ncbi.nlm.nih.gov/pubmed?term=Lee S%5BAuthor%5D&cauthor=true&cauthor_uid=22535926), [Um Y](http://www.ncbi.nlm.nih.gov/pubmed?term=Um Y%5BAuthor%5D&cauthor=true&cauthor_uid=22535926), [Oh MK](http://www.ncbi.nlm.nih.gov/pubmed?term=Oh MK%5BAuthor%5D&cauthor=true&cauthor_uid=22535926), [Kim YR](http://www.ncbi.nlm.nih.gov/pubmed?term=Kim YR%5BAuthor%5D&cauthor=true&cauthor_uid=22535926), [Lee J](http://www.ncbi.nlm.nih.gov/pubmed?term=Lee J%5BAuthor%5D&cauthor=true&cauthor_uid=22535926), [Yang KS](http://www.ncbi.nlm.nih.gov/pubmed?term=Yang KS%5BAuthor%5D&cauthor=true&cauthor_uid=22535926): **Complete genome sequence of the 2,3-butanediol-producing *Klebsiella pneumoniae* strain KCTC 2242.** [*J Bacteriol*](http://www.ncbi.nlm.nih.gov/pubmed/?term=10.1128%2FJB.00027-12) 2012, **10**:2736-7.

# 19. [**Wu KM**](http://www.ncbi.nlm.nih.gov/pubmed?term=Wu KM%5BAuthor%5D&cauthor=true&cauthor_uid=19447910), [**Li LH**](http://www.ncbi.nlm.nih.gov/pubmed?term=Li LH%5BAuthor%5D&cauthor=true&cauthor_uid=19447910), [**Yan JJ**](http://www.ncbi.nlm.nih.gov/pubmed?term=Yan JJ%5BAuthor%5D&cauthor=true&cauthor_uid=19447910), [**Tsao N**](http://www.ncbi.nlm.nih.gov/pubmed?term=Tsao N%5BAuthor%5D&cauthor=true&cauthor_uid=19447910), [**Liao TL**](http://www.ncbi.nlm.nih.gov/pubmed?term=Liao TL%5BAuthor%5D&cauthor=true&cauthor_uid=19447910), [**Tsai HC**](http://www.ncbi.nlm.nih.gov/pubmed?term=Tsai HC%5BAuthor%5D&cauthor=true&cauthor_uid=19447910), [**Fung CP**](http://www.ncbi.nlm.nih.gov/pubmed?term=Fung CP%5BAuthor%5D&cauthor=true&cauthor_uid=19447910), [**Chen HJ**](http://www.ncbi.nlm.nih.gov/pubmed?term=Chen HJ%5BAuthor%5D&cauthor=true&cauthor_uid=19447910), [**Liu YM**](http://www.ncbi.nlm.nih.gov/pubmed?term=Liu YM%5BAuthor%5D&cauthor=true&cauthor_uid=19447910), [**Wang JT**](http://www.ncbi.nlm.nih.gov/pubmed?term=Wang JT%5BAuthor%5D&cauthor=true&cauthor_uid=19447910), [**Fang CT**](http://www.ncbi.nlm.nih.gov/pubmed?term=Fang CT%5BAuthor%5D&cauthor=true&cauthor_uid=19447910), [**Chang SC**](http://www.ncbi.nlm.nih.gov/pubmed?term=Chang SC%5BAuthor%5D&cauthor=true&cauthor_uid=19447910), [**Shu HY**](http://www.ncbi.nlm.nih.gov/pubmed?term=Shu HY%5BAuthor%5D&cauthor=true&cauthor_uid=19447910), [**Liu TT**](http://www.ncbi.nlm.nih.gov/pubmed?term=Liu TT%5BAuthor%5D&cauthor=true&cauthor_uid=19447910), [**Chen YT**](http://www.ncbi.nlm.nih.gov/pubmed?term=Chen YT%5BAuthor%5D&cauthor=true&cauthor_uid=19447910), [**Shiau YR**](http://www.ncbi.nlm.nih.gov/pubmed?term=Shiau YR%5BAuthor%5D&cauthor=true&cauthor_uid=19447910), [**Lauderdale TL**](http://www.ncbi.nlm.nih.gov/pubmed?term=Lauderdale TL%5BAuthor%5D&cauthor=true&cauthor_uid=19447910), [**Su IJ**](http://www.ncbi.nlm.nih.gov/pubmed?term=Su IJ%5BAuthor%5D&cauthor=true&cauthor_uid=19447910), [**Kirby R**](http://www.ncbi.nlm.nih.gov/pubmed?term=Kirby R%5BAuthor%5D&cauthor=true&cauthor_uid=19447910), [**Tsai SF**](http://www.ncbi.nlm.nih.gov/pubmed?term=Tsai SF%5BAuthor%5D&cauthor=true&cauthor_uid=19447910): [Genome sequencing and comparative analysis of *Klebsiella pneumoniae* NTUH-K2044, a strain causing liver abscess and meningitis](http://www.ncbi.nlm.nih.gov/pmc/articles/PMC2704730/). *J Bacteriol* 2009, 191: 4492–501.

20. [Liu P](http://www.ncbi.nlm.nih.gov/pubmed?term=Liu P%5BAuthor%5D&cauthor=true&cauthor_uid=22408243), [Li P](http://www.ncbi.nlm.nih.gov/pubmed?term=Li P%5BAuthor%5D&cauthor=true&cauthor_uid=22408243), [Jiang X](http://www.ncbi.nlm.nih.gov/pubmed?term=Jiang X%5BAuthor%5D&cauthor=true&cauthor_uid=22408243), [Bi D](http://www.ncbi.nlm.nih.gov/pubmed?term=Bi D%5BAuthor%5D&cauthor=true&cauthor_uid=22408243), [Xie Y](http://www.ncbi.nlm.nih.gov/pubmed?term=Xie Y%5BAuthor%5D&cauthor=true&cauthor_uid=22408243), [Tai C](http://www.ncbi.nlm.nih.gov/pubmed?term=Tai C%5BAuthor%5D&cauthor=true&cauthor_uid=22408243), [Deng Z](http://www.ncbi.nlm.nih.gov/pubmed?term=Deng Z%5BAuthor%5D&cauthor=true&cauthor_uid=22408243), [Rajakumar K](http://www.ncbi.nlm.nih.gov/pubmed?term=Rajakumar K%5BAuthor%5D&cauthor=true&cauthor_uid=22408243), [Ou HY](http://www.ncbi.nlm.nih.gov/pubmed?term=Ou HY%5BAuthor%5D&cauthor=true&cauthor_uid=22408243): **Complete genome sequence of *Klebsiella pneumoniae* subsp. *pneumoniae* HS11286, a multidrug-resistant strain isolated from human sputum.** [*J Bacteriol*](http://www.ncbi.nlm.nih.gov/pubmed/?term=10.1128%2FJB.00043-12.) 2012, **7**:1841-2.
